# Comparative Proteomic Analysis of the Graft Unions in Hickory (*Carya cathayensis*) Provides Insights into Response Mechanisms to Grafting Process

**DOI:** 10.3389/fpls.2017.00676

**Published:** 2017-04-27

**Authors:** Dongbin Xu, Huwei Yuan, Yafei Tong, Liang Zhao, Lingling Qiu, Wenbin Guo, Chenjia Shen, Hongjia Liu, Daoliang Yan, Bingsong Zheng

**Affiliations:** ^1^State Key Laboratory of Subtropical Silviculture, Zhejiang A&F UniversityLinan, China; ^2^Center for Cultivation of Subtropical Forest Resources, Zhejiang A&F UniversityLinan, China; ^3^College of Life and Environmental Sciences, Hangzhou Normal UniversityHangzhou, China; ^4^Crop and Nuclear Technology Institute, Zhejiang Academy of Agricultural SciencesHangzhou, China

**Keywords:** flavonoid biosynthesis, graft, hickory, TMT, secondary metabolism

## Abstract

Hickory (*Carya cathayensis*), a tree with high nutritional and economic value, is widely cultivated in China. Grafting greatly reduces the juvenile phase length and makes the large scale cultivation of hickory possible. To reveal the response mechanisms of this species to grafting, we employed a proteomics-based approach to identify differentially expressed proteins in the graft unions during the grafting process. Our study identified 3723 proteins, of which 2518 were quantified. A total of 710 differentially expressed proteins (DEPs) were quantified and these were involved in various molecular functional and biological processes. Among these DEPs, 341 were up-regulated and 369 were down-regulated at 7 days after grafting compared with the control. Four auxin-related proteins were down-regulated, which was in agreement with the transcription levels of their encoding genes. The Kyoto Encyclopedia of Genes and Genomes (KEGG) analysis showed that the ‘Flavonoid biosynthesis’ pathway and ‘starch and sucrose metabolism’ were both significantly up-regulated. Interestingly, five flavonoid biosynthesis-related proteins, a flavanone 3-hyfroxylase, a cinnamate 4-hydroxylase, a dihydroflavonol-4-reductase, a chalcone synthase, and a chalcone isomerase, were significantly up-regulated. Further experiments verified a significant increase in the total flavonoid contents in scions, which suggests that graft union formation may activate flavonoid biosynthesis to increase the content of a series of downstream secondary metabolites. This comprehensive analysis provides fundamental information on the candidate proteins and secondary metabolism pathways involved in the grafting process for hickory.

## Introduction

Hickory (*Carya cathayensis*), an important member of the *Juglandaceae*, is a popular and commercially available tree that is widely grown in Zhejiang Province, China. Hickory seeds contain about 70% oil and have considerable amounts of nutritious components, such as polyunsaturated fatty acids (Ni and Shi, 2014; Huang et al., 2016; Wang et al., 2017). When growing outside of cultivation, the long juvenile stage lasts for over 10 years before a hickory tree reaches maturity and reduces yields (Wang et al., 2014; Sima et al., 2015). Grafting an ancient technique plays an important role in plant propagation and improvement (Pina and Errea, 2005; Zheng et al., 2010). In cultivation, the most frequently consumed horticultural plants, including tomato, cucumber, and melon, are often grafted (Estan et al., 2005; Sigüenza et al., 2005; Zhou et al., 2009). Grafting provides a good method for reducing the length of the juvenile stage during the cultivation of hickory (Zheng et al., 2010).

The formation of callus tissue at the graft interface initiates the grafting process, and a lack of callus formation leads to grafting failure (Moore and Walker, 1983). Once the callus has formed, a series of protein molecules are released from the plasmalemmas to form a complex with catalytic activity (Pina and Errea, 2005). Grafting success primarily depends on the survival rate of the grafted material and the symbiotic relationship between the rootstock and the scion during the growth period. Graft compatibility between the rootstock and scion is a major factor affecting the survival of grafted plants (Leonardi and Romano, 2004). However, the mechanism underlying the grafting-induced physiological processes is largely unknown.

Recently, many transcriptomic and proteomic studies have investigated the biochemical and physiological responses in various plant species that occur at the early phase in response to grafting. In watermelon, transcriptome and digital gene expression profiling data have provided new insights into the grafting-responsive mRNAs that are involved in many different biological and metabolic processes (Liu et al., 2015). In grapevine, the formation of a graft union induced transcriptional changes related to various processes, including wounding responses, cell wall establishment, hormone signaling, and secondary metabolism (Cookson et al., 2013). A proteomic analysis revealed that higher expressions of key enzymes involved in metabolic systems, including the Calvin cycle, amino acids biosynthesis, carbohydrate and energy metabolism, contributed to the salt tolerance of rootstock-grafted watermelon (Yang et al., 2012). Different scion-rootstock combinations may control susceptibility to *Fusarium oxysporum* f. sp. *radicis-lycopersici* of tomato. Proteomic analysis also showed that the accumulation of specific proteins belonging to three main broad classes, including the components involved in stress response, carbohydrate metabolism, protein turnover, is involved in eliciting resistance to *F. oxysporum* f. sp. *radicis-lycopersici* infection (Vitale et al., 2014). Muneer’s proteomic study demonstrated the responses of grafted tomato to various temperatures (Muneer et al., 2016). In populous, the expression of many proteins, including photosynthetic proteins, ion binding/transport proteins, and cellular defense proteins, were regulated in graft unions under different temperature conditions (Lomaglio et al., 2015).

Previous studies of hickory have focused primarily on the morphological, physiological and transcriptional changes that occur during the grafting process (Zheng et al., 2002; Jin et al., 2011; Wang et al., 2012, 2015; Huang et al., 2013, 2015; Li et al., 2014; Shen et al., 2014; Ji et al., 2016). However, no proteomic data have been reported to date in hickory. In this study, we employed a proteomics-based approach to identify differentially expressed proteins (DEPs) during the hickory grafting process. The results provide useful information about how to improve the survival rate of grafted hickory.

## Materials and Methods

### Experiment Design, Plant Materials, and Protein Extraction

Hickory (*Carya cathayensis* Sarg.) trees were been planted in a greenhouse at the campus of Zhejiang A & F University (Lin’an, China) at a temperature of 25 ± 1°C with a light/dark cycle of 12/12 h and 60–70% relative humidity. All hickory plants were watered once a week. A nutrient solution containing 1.425 mM NH_4_NO_3_, 0.323 mM NaH_2_PO_4_, 0.513 mM K_2_SO_4_, 0.998 mM CaCl_2_, 1.643 mM MgSO_4_, 0.009 mM MnCl_2_, 0.075 mM (NH_4_)_6_Mo_7_O_24_, 0.019 mM H_3_BO_3_, 0.155 mM CuSO_4_, 1 mM FeCl_3_, 0.070 mM citric acid and 0.152 mM ZnSO_4_ was used in our study. The grafting experiment was carried out at March, 2015.

Hickory samples were collected from graft unions (the stem segment that includes the swollen part of the rootstock and the scions) at 7 days after grafting. The detail information of sampling has been showed in Supplementary Figure S1. A mixture sample taken from 2-year-old rootstock and a 1-year-old scion before grafting was used as the control. The samples were first ground in liquid nitrogen, then transferred to 5 mL centrifuge tubes, and sonicated three times on ice with pre-cooled lysis buffer (8 M urea, 2 mM ethylenediaminetetraacetic acid, 10 mM dithiothreitol and 1% Protease Inhibitor Cocktail VI). The remaining debris was removed by centrifugation at 20,000 *g* and 4°C for 10 min. Then, the protein was precipitated with pre-cooled 15% TCA for 2 h at -20°C. The supernatant was discarded after 3 min of centrifugation at 20,000 *g* under 4°C. Finally, the remaining precipitate was washed three times with cold acetone buffer. The protein was re-dissolved in a buffer (8 M urea, 100 mM tetraethylammonium bromide, pH 8.0), and a 2-D Quant kit (GE Healthcare, Pittsburgh, PA, USA) was used to determine the protein concentration according to the manufacturer’s instructions.

### Trypsin Digestion and Tandem Mass Tag (TMT) Labeling

First, the protein solution was reduced with 10 mM dithiothreitol for 1 h at 37°C and alkylated with 20 mM iodoacetamide for 45 min at room temperature in the dark. The protein samples were digested with Trypsin Gold (Promega, Madison, WI, USA) to produce the trypsin digestion samples. The trypsin was added at a mass ratio of 1:50 trypsin:protein for the first overnight digestion and at 1:100 trypsin: protein for the second 4 h digestion. Approximately 100 μg protein of each sample was digested with trypsin.

After trypsin digestion, the peptide was desalted by a Strata X C18 SPE column (Phenomenex, Torrance, CA, USA) and vacuum-dried. The peptide was reconstituted in 0.5 M TEA buffer and processed using a 6-plex TMT kit according to the manufacturer’s protocol (Thermo-Scientific, Rockford, IL, USA). Briefly, one unit of TMT reagent (defined as the amount of reagent required to label 100 μg of protein) was thawed and reconstituted in 24 μL ACN. The peptide mixtures were then incubated for 2 h at room temperature and pooled, desalted, and dried by vacuum centrifugation.

### High Performance Liquid Chromatography (HPLC) Fractionation

The sample was then fractionated into fractions by high pH reverse-phase HPLC using an Agilent 300Extend C18 column (Agilent, Santa Clara, CA, USA) (5 μm particles, 4.6 mm ID). The wavelength 250 nm is used for detection of peptides. Briefly, the peptides were first separated into 80 fractions over 80 min using a gradient of 2–60% ACN in 10 mM ammonium bicarbonate (pH 10). Then, the peptides were combined into 18 fractions and dried by vacuum centrifuging. A representative HPLC data has been showed in Supplementary Figure S2.

### Liquid Chromatography (LC)-Tandem Mass Spectrometry (MS/MS) Analysis

The peptides were dissolved in 0.1% formic acid (FA) buffer and directly loaded onto a reversed-phase Agilent 300Extend C18 column (Agilent, Santa Clara, CA, USA). Then the peptides were separated by a reversed-phase analytical column (Acclaim PepMap RSLC, Thermo Scientific). The gradient was an increase from 6 to 22% solvent B (0.1% FA in 98% ACN) over 26 min, 22–35% for 8 min, and then rose to 80% over 3 min. This was held at 80% for the last 3 min. The constant flow rate was 400 nL/min on an EASY-nLC 1000 UPLC system. The resulting peptides were analyzed using a Q ExactiveTM hybrid quadrupole-Orbitrap mass spectrometer (Thermo-Fisher Scientific, Shanghai, China).

The peptides were subjected to an nanospray ionization source followed by MS/MS in a Q ExactiveTM (Thermo-Fisher Scientific, Shanghai, China) coupled to the UPLC online. The intact peptides were detected in the Orbitrap mass spectrometer at a resolution of 70,000. The peptides were selected for MS/MS using a NCE setting of 28 and the ion fragments were detected in the Orbitrap at a resolution of 17,500. A data-dependent procedure that alternated between one MS scan followed by 20 MS/MS scans was applied to the top 20 precursor ions above a threshold ion count of 1E4 in the MS survey scan with a 30 s dynamic exclusion. The electrospray voltage applied was 2.0 kV. Automatic gain control was used to prevent overfilling of the ion trap and 5E4 ions were accumulated to generate the MS/MS spectra. The m/z scan range was 350–1800 for the MS scans and the fixed first mass was set at 100 m/z.

The mass spectrometry proteomics data have been deposited to the Proteome EXchange Consortium via the PRIDE partner repository with the dataset identifier PXD006025.

### Database Search

The resulting MS/MS data were processed using the Mascot search engine (v.2.3.0)^1^. Tandem mass spectra were searched against a published transcriptome data, which has been up-loaded to database by our lab (NCBI Sequence Read Archive database under the accession numbers SRX2576694">SRX2576694), concatenated with reverse decoy database (Qiu et al., 2016). Trypsin/P was specified as a cleavage enzyme and up to 2 missing cleavages were allowed. The mass error was set to 0.02 Da for the precursor ions and fragment ions. Carbamidomethyl on Cys was specified as a fixed modification and oxidation on Met was specified as a variable modification. TMT-6-plex was selected in Mascot for protein quantification. False discovery rate (FDR) thresholds for peptide and protein identification were specified at 1%. The peptide ion score was set to ≥20.

The quantitative value of the unique peptide was calculated according to the ratio of the ion signal intensity in secondary spectrum. Then, the mean value of all unique peptides that related to each protein was used to quantify to protein expression.

### Annotation Methods

Annotation was performed as described before (Shen et al., 2016). In brief, gene ontology (GO) annotation of our proteome was derived from the UniProt-GOA database^2^. Firstly, all identified protein IDs were converted to UniProt IDs and mapped to GO IDs. Then, InterProScan soft was used to annotate the proteins that were not annotated by UniProt-GOA database based on protein sequence alignment method. Lastly, all proteins were classified by GO annotation into three categories: biological process, cellular component and molecular function.

Kyoto Encyclopedia of Genes and Genomes database^3^ was used to annotate protein pathways. Firstly, KEGG online service tools KAAS^4^ was used to annotate the KEGG description of proteins. Then, the annotation results were mapped to the KEGG pathway database using online tool KEGG Mapper^5^.

Protein domain functional description was annotated by InterPro domain database^6^. InterPro is a database that integrates diverse information about protein families, domains and functional sites, and makes it freely available to the public via Web-based interfaces and services.

Lastly, the quantified proteins in this study were divided into four quantitative category according to the quantification ratio to generated four quantitative categories: Q1 (0 < D7/D0 Ratio < 1/1.5), Q2 (1/1.5 < D7/D0 Ratio < 1/1.3), Q3 (1.3 < D7/D0 Ratio < 1.5) and Q4 (D7/D0 Ratio > 1.5).

### Enrichment Analysis

For each GO category, a two-tailed Fisher’s exact test was employed to determine the enrichment of all DEPs found. Correction for multiple hypothesis testing was carried out using standard FDR control methods. The GO with a corrected *p*-value < 0.05 is considered significant. The MeV software was used for K-means cluster.

For each KEGG term, a two-tailed Fisher’s exact test was employed to test the enrichment of the DEPs. Correction for multiple hypothesis testing was carried out using standard FDR control methods. The pathway with a corrected *p*-value < 0.05 was considered significant.

For each category protein, InterPro (a resource that provides functional analysis of protein sequences by classifying them into families and predicting the presence of domains and important sites) database was searched. A two-tailed Fisher’s exact test was employed to test the enrichment of the DEPs. Correction for multiple hypothesis testing was carried out using standard FDR control methods and domains with a corrected *p*-value < 0.05 was considered significant. For the bioinformatics analysis, such as the GO-base and KEGG-base enrichment, all the sequences in the database were used as the background (Shen et al., 2017).

There were significant differences in the expression levels of the DEPs with different functions. In order to meet the requirements of the hierarchical clustering method, the *P*-value was transformed into *Z*-score after log transformation (Shen et al., 2016).

Formula:

$$\begin{array}{c} Z\;sample-i\;=\;\frac{\log2(\mathrm{Signalsample}\;-\;i)\;-\mathrm{Mean}\;(Log2\;(\mathrm{Signal})\;\mathrm{of}\;\mathrm{all}\;\mathrm{samples})}{\mathrm{Standard}\mathrm{deviation}(Log2\;(\mathrm{Signal})\;\mathrm{of}\mathrm{all}\mathrm{samples})} \end{array}$$

### Determination of Total Flavonoids by HPLC

The standard rutin was purchased from National Institute for the Control of Pharmaceutical and Biological Products (ID: 100080-200707). A total of 200.0 mg/L of the rutin standard solution was prepared by dissolving the rutin reference material in 70% ethanol.

Each dried plant sample was crushed. The extraction and HPLC analysis of flavonoids of the samples were carried out as described before with slight modification (Xu et al., 2012). Briefly, frozen samples were extracted with 30 μl extraction buffer (methanol: acetate: H_2_O = 9:1:10) per 1 mg dry samples at 37°C 30 min. The supernatant was filtered by a 0.25 μm filter membrane after centrifugation at 14000 *g*. Then, 1 mL supernatant was applied to waters HPLC e2695 series. HPLC was carried out by a XBridge C18 (Φ4.6 mm × 250 mm) at flow rate of 0.5 mL/min. Elution gradient with solvent A [CH_3_CN-H_2_O-TFA (10:90:0.1)] and solvent B [CH_3_CN-H_2_O-TFA (90:10:0.1)] and the following elution profile (0 min 100% A, 30 min 70% A, 32 min 0% A, 33 min 0% A, 35 min 100% A) using linear gradients between the time points. Flavonoids were detected at 360 nm (Feng et al., 2013).

#### IAA Content Measurement

Plant samples were harvested from grafting union at 0 and 7 days after grafting. All samples were homogenized by 50 mM Tris-HCl buffer, pH 7.6 and were collected by centrifugation at 12,000 *g* in a 1.5 ml centrifuge. The supernatants were used for further purify. Three independent biological replicates of 20 mg each were purified after addition of 250 pg of 13C_6_-IAA internal standard using ProElu C18.^7^ Auxin content were measured with FOCUS GC-DSQII (Thermo Fisher Scientific Inc., Austin, TX, USA) (Shen et al., 2015).

### Statistical Analysis

Significant differences between different samples were calculated using a one-way analysis of variance with a Tukey’s test (at a significance level of α = 0.01) in Excel software. All of the expression analyses were performed for three biological replicates. All reported values represent the averages of three replicates, and data are expressed as the mean plus or minus the standard deviation (mean ± SD).

## Results

### Quantitative Proteome Analysis and Quality Control Validation of the MS Data

**FIGURE 1:**
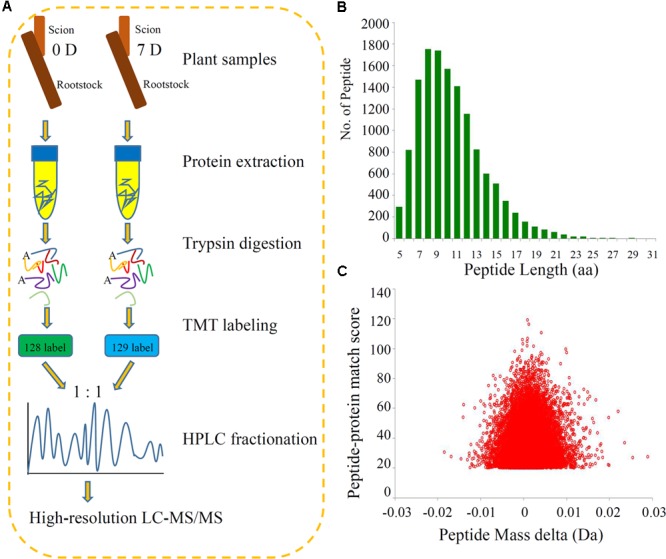
**Experimental strategy for the quantitative proteome analysis and QC validation of MS data. (A)** Proteins were extracted in three biological replicates per sample and trypsin digested. The resulting peptides were directly analyzed by HPLC-MS/MS. **(B)** Length distribution of all identified peptides. **(C)** Mass delta of all identified peptides.

We identified the DEPs during the process of grafting hickory by applying high-throughput quantitative proteomics and TMT isobaric labeling (**Figure 1A**). A large number of peptides were identified based on the MS data. We checked the mass error of all the identified peptides. The distribution of the mass errors was near zero and most mass errors were less than 0.02 Da, which means that the mass accuracy of the MS data fitted the requirements (**Figure 1B**). Most peptides were between 8 and 16 amino acids long, which agreed with the known properties of tryptic peptides. Our data suggested that sample preparation met the required standards (**Figure 1C**).

Integration of the basic HPLC fractionation and LC-MS/MS data allowed the identification of 3723 proteins, of which 2518 proteins were quantified, and these peptides exhibited distinct abundances depending on their lengths. We annotated the proteins using several different categories, including GO, domain, pathway, and subcellular localization, to further understand the functions and features of the identified and quantified proteins. Information about the identified and quantified proteins is shown in Supplementary Table S1.

### Impacts of Grafting on Global Proteome Levels in Hickory

Further analysis identified 710 proteins as DEPs during the hickory grafting process. Among the DEPs, 341 proteins were up-regulated and 369 proteins were down-regulated at 7 days after grafting compared with the control (Supplementary Table S2 and Figure S2). Mortalin-like protein 28-like protein, chlorophyll a/b-binding protein, predicted protein, lysine histidine transporter 1-like protein, and an unnamed protein product, were up-regulated over fivefold by grafting compared with the control. Only one protein, a GRAS family transcription factor, was down-regulated over fivefold.

**FIGURE 2:**
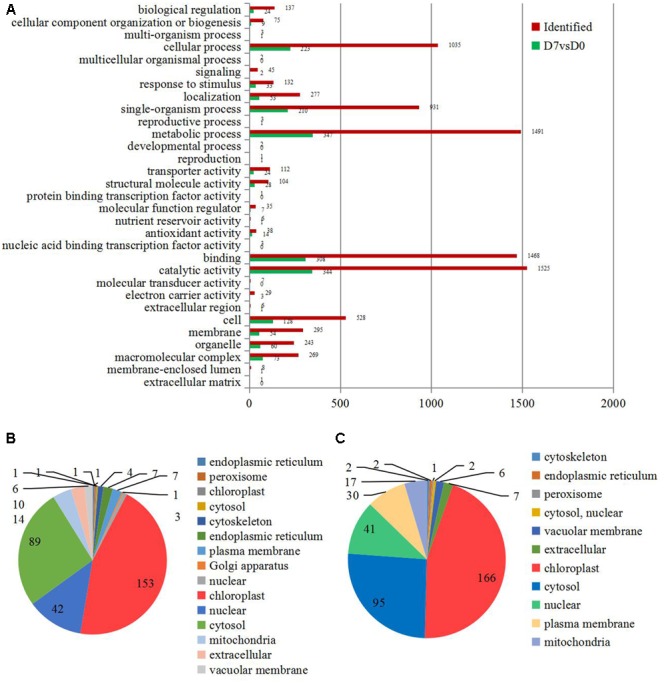
**The information of the concerning DEPs during the graft process. (A)** All DEPs during the graft process were classified by GO terms based on their cellular component, molecular function, and biological process. Subcellular classify of up-regulated DEPs **(B)** and down-regulated DEPs **(C)**.

All identified proteins and DEPs found during the grafting process were classified using GO terms based on the cellular component, molecular function, and biological process categories. The numbers of identified proteins and DEPs in each GO term are shown in **Figure 2A**. In detail, 41% of the identified proteins (1525) and 13.7% of the DEPs (344) have catalytic activities; about 40% of the identified proteins (1491) and 13.8% of the DEPs (347) were involved in metabolic processes; and 39.4% of the identified proteins (1468) and 12.2% of the DEPs (308) were annotated as ‘binding proteins’ (**Figure 2A**).

The DEPs were also classified according to their subcellular locations. For the up-regulated proteins, a total of 14 subcellular components were identified, including chloroplast (45.00%), cytosol (26.18%), nucleus (12.35%), mitochondria (4.12%), and extracellular areas (2.94%) (**Figure 2B**). Only 11 subcellular components were identified for the down-regulated proteins. These included chloroplast (44.98%), cytosol (25.75%), nucleus (11.11%), plasma membrane (8.13%), and mitochondria (4.61%) (**Figure 2C**).

### Enrichment Analysis of the DEPs in Hickory during the Grafting Process

**FIGURE 3:**
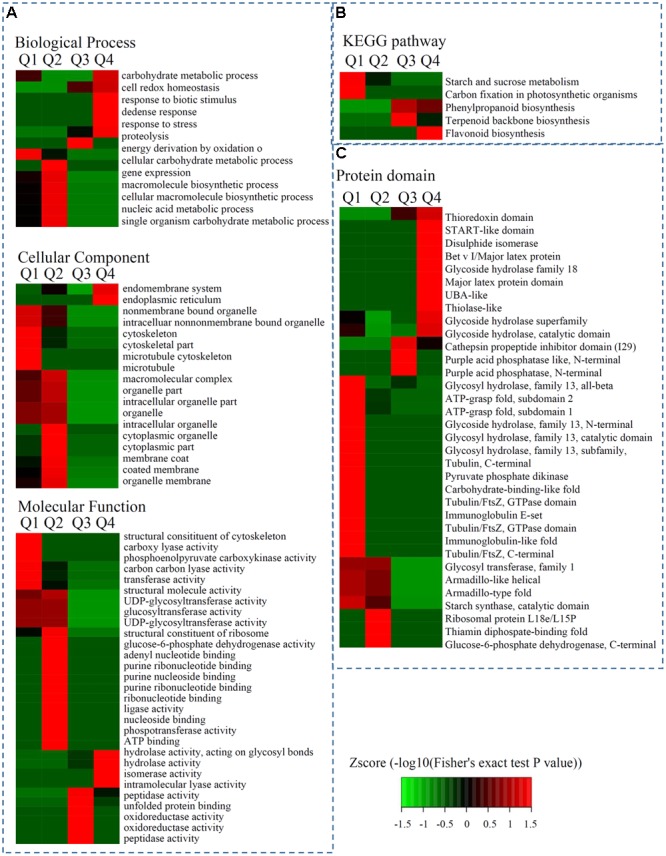
**Enrichment analysis of DEPs in hickory during grafting process. (A)** Significantly enriched GO terms of DEPs concerning cellular component, molecular function and biological process. **(B)** Significantly enriched KEGG terms of DEPs. **(C)** Significantly enriched protein domains of DEPs.

The significantly enriched cellular component GO terms were mainly associated with ‘bounding membrane of organelles’ (*p* = 0.0061), ‘endomembrane system’ (*p* = 0.0074), and ‘endoplasmic reticulum’ (*p* = 0.0250). The top five significantly enriched molecular function GO terms were ‘UDP-glycosyltransferase activity’ (*p* = 0.0099), ‘glucosyltransferase activity’ (*p* = 0.0099), ‘UDP-glucosyltransferase activity’ (*p* = 0.0099), ‘NADP binding’ (*p* = 0.0212), and ‘heme binding’ (*p* = 0.0386). The majority of the proteins categorized in the biological process associated GO terms were classified into the ‘response to stress’ (*p* = 0.0034), ‘response to oxygen-containing compounds’ (*p* = 0.0263), ‘response to acid chemicals’ (*p* = 0.0384), and ‘response to water’ (*p* = 0.0384) terms (**Figure 3A** and Supplementary Table S3).

The biological functions of the DEPs were identified by analyzing the proteins with greater than 1.3-fold changes between two samples using enrichment-based clustering and KEGG pathway analysis. The results revealed that the DEPs (7D/0D < 1/1.5) were most strongly associated with ‘starch and sucrose metabolism,’ and ‘carbon fixation in photosynthetic organisms’; the 7D/0D > 1.3 DEPs were most strongly involved in phenylpropanoid biosynthesis and terpenoid backbone biosynthesis; and the 7D/0D > 1.5 DEPs were associated with flavonoid biosynthesis (**Figure 3B**).

Protein domain analysis revealed that the up-regulated DEPs (Q4: 7D/0D > 1.5) were highly enriched in ‘Bet v I/Major latex protein,’ ‘START-like domain,’ ‘Glycoside hydrolase,’ and ‘Glycoside hydrolase superfamily’ proteins. Meanwhile, the down-regulated DEPs (Q1: 7D/0D < 1/1.5) were highly enriched in ‘Immunoglobulin-like fold,’ ‘Glycoside hydrolase, family 13, N-terminal’, ‘Immunoglobulin E-set,’ and ‘Glycosyl hydrolase, family 13, subfamily, catalytic domain’ proteins (**Figure 3C** and Supplementary Table S4).

### Comparative Transcriptomics and Proteomics of Hickory during the Grafting Process

**FIGURE 4:**
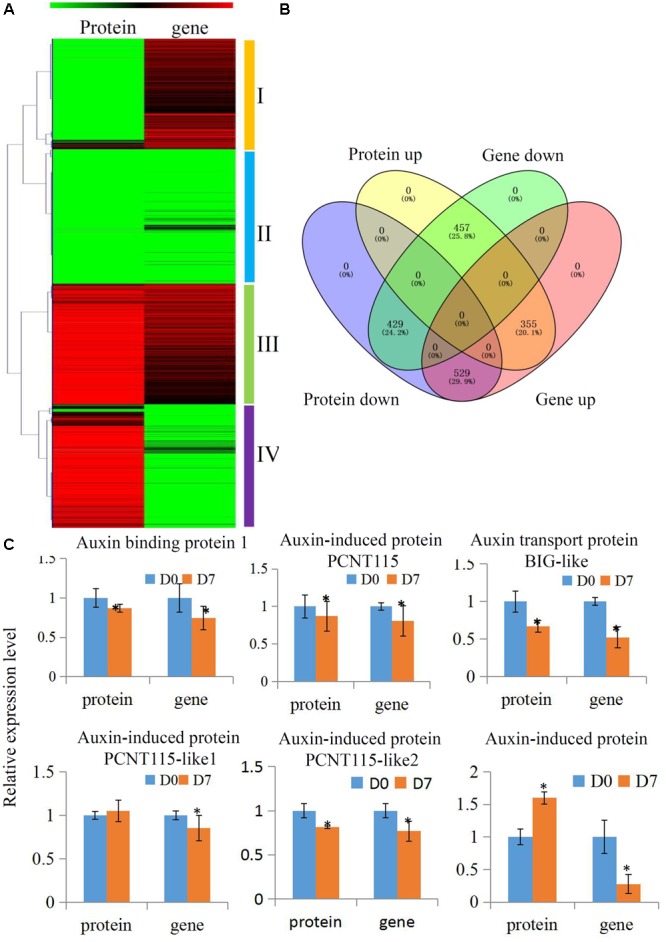
**Comparative transcriptomics and proteomics of hickory during grafting process. (A)** Heat map for cluster analysis of the differentially expressed proteins combining with their transcripts by K-means method. Red indicates up-regulation and green indicates down-regulation. Proteins and transcripts were grouped into four groups based abundance values as follows: (I) proteins down-regulated and transcripts up-regulated; (II) proteins down-regulated and transcripts down-regulated; (III) proteins up-regulated and transcripts up-regulated; and (IV) proteins up-regulated and transcripts down-regulated. **(B)** Distinct expression regulation of proteins and transcripts was showed by a Venn Diagram. **(C)** Relative expression level of several auxin pathway proteins of hickory during grafting process. Significant differences in expression level were indicated by “^∗^”.

Many biological processes can be precisely regulated at both the mRNA and protein levels. To delineate how the grafting process that is modulated at different levels of abundance for mRNA and proteins, we performed a combined hierarchical clustering of the identified proteins and their encoding genes. We searched for these protein identifications (IDs) in our transcriptome and most of the quantified proteins (1770 out of 2518) were successfully identified (Supplementary Table S5). We categorized proteins into four abundance groups: I, II, III, and IV. Class I proteins were down-regulated and their encoding genes were up-regulated. In Class II, both proteins and their encoding genes were down-regulated. In Class III, both proteins and their encoding genes were up-regulated. In Class IV, proteins were up-regulated and their encoding genes were down-regulated (**Figures 4A,B**).

### Changes to Auxin Signaling Pathway Proteins during the Grafting Process

Auxin plays essential roles in the formation of the callus during vascular tissue and vein development (Wetmore and Rier, 1963). In our study, several auxin-related protein expressions were analyzed to uncover how the auxin signaling pathway was involved in the grafting process of hickory. In total, six auxin signaling pathway-related proteins, including an auxin response 4-like protein, an auxin-induced protein PCNT115, an auxin transport protein BIG-like, an auxin-induced protein PCNT115-like, and an auxin-induced in root cultures protein, were identified and quantified in our hickory proteomic data. We also searched the IDs for these proteins in our transcriptome and all of them were identified (**Figure 4C** and Supplementary Table S6). Among these proteins, four proteins were significantly down-regulated, which was in agreement with the transcription levels of their encoding genes. Furthermore, the auxin content has been measured during the grafting process. Our data showed that the endogenous auxin contents were up-regulated significantly in grafting union (Supplementary Figure S3).

### Involvement of Flavonoid Biosynthesis in the Hickory Grafting Process

**FIGURE 5:**
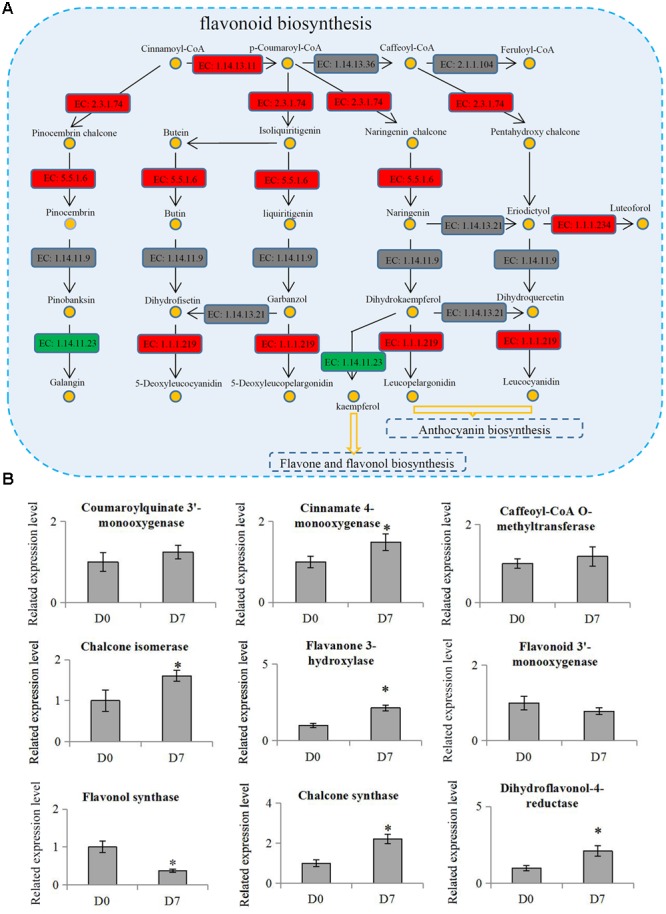
**Involvement of flavonoid biosynthesis in the grafting process in hickory. (A)** Schematic representation of the proteins involved in flavonoid biosynthesis pathway. ‘EC: 1.14.13.11’: trans-cinnamate 4-monooxygenase; ‘EC 1.14.13.36’: 5-*O*-(4-coumaroyl)-D-quinate 3′-monooxygenase; ‘EC: 2.1.1.104’: caffeoyl-CoA 3-*O*-methyltransferase; ‘EC: 2.3.1.74’: naringenin-chalcone synthase; ‘EC: 5.5.1.6’: chalcone isomerase; ‘EC: 1.14.11.9’: flavanone 3-dioxygenase; ‘EC: 1.14.13.21’: flavonoid 3′-monooxygenase; ‘EC: 1.1.1.234’: flavanone 4-reductase; ‘EC: 1.14.11.23’: flavonol synthase; ‘EC: 1.1.1.219’: dihydrokaempferol 4-reductase. Red indicates up-regulation proteins, green indicates down-regulation proteins and gray indicates un-identified proteins. **(B)** Relative expression levels of flavonoid biosynthesis related proteins in hickory. Significant differences in expression level were indicated by “∗”.

The KEGG results showed that two metabolism pathways, ‘Starch and sucrose metabolism’ and ‘Carbon fixation in photosynthetic organisms,’ were significantly down-regulated, but three other metabolism pathways, ‘Phenylpropanoid biosynthesis,’ ‘Terpenoid backbone biosynthesis,’ and ‘Flavonoid biosynthesis,’ were significantly up-regulated. Flavonoids are secondary metabolites in plants that were regulated by the phenylpropanoid pathway (Buer et al., 2008). The flavonoid biosynthesis pathway (*p* = 0.0009) was the most significantly differential pathway during the hickory grafting process (**Figure 5A**). The flavonoid biosynthesis pathway has been identified in various plant species, which gave us an opportunity to identify flavonoid-related proteins in hickory. In total, 10 flavonoid biosynthesis-related proteins were identified and nine proteins were quantified in hickory. Among these quantified proteins, five proteins: a flavanone 3-hyfroxylase, a cinnamate 4-hydroxylase, a dihydroflavonol-4-reductase, a chalcone synthase, and a chalcone isomerase, were significantly up-regulated. Only one protein, a flavonol synthase, was significantly down-regulated (**Figure 5B**).

**FIGURE 6:**
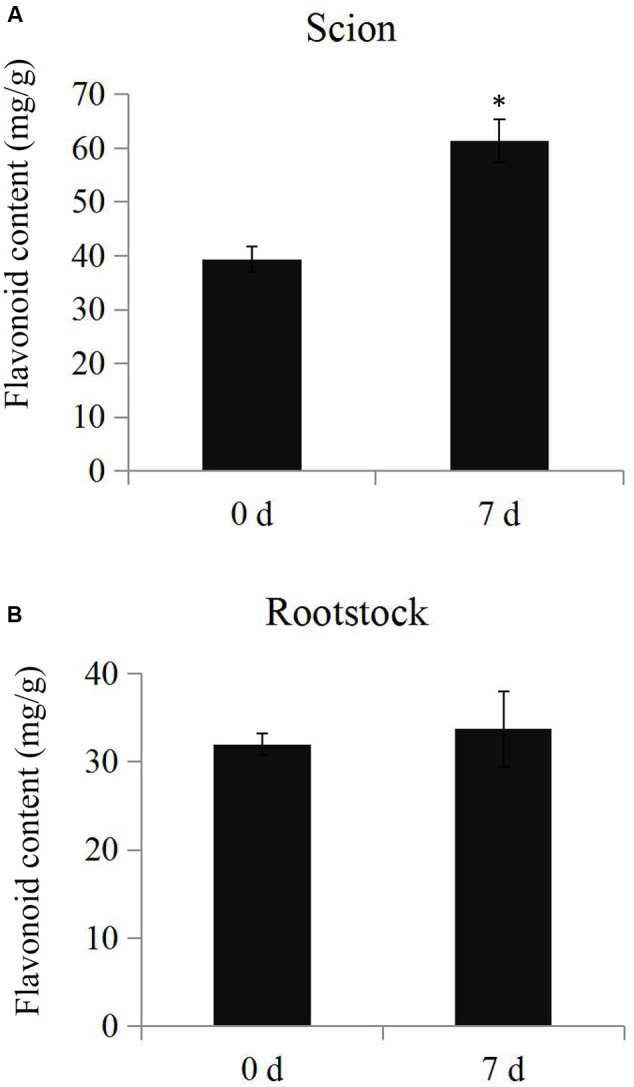
**Determination of total flavonoid contents.** The total flavonoid contents in the scion **(A)** and rootstock **(B)** at time points 0 and 7 days during the grafting process. Significant differences in the total flavonoid contents were indicated by “∗”.

The total flavonoid content was determined in the samples from scions and rootstocks during the hickory grafting process. The data showed that total flavonoid content in the scions significantly increased from 35.3 mg.g^-1^ to 51.4 mg.g^-1^ after grafting, but no significant differences in total flavonoid content were observed in the rootstock samples (**Figure 6**). The number of the up-regulated flavonoid biosynthesis-related proteins was larger than the down-regulated proteins, which helped to explain the increase in the total flavonoid content (Supplementary Figure S3).

## Discussion

Hickory (*Carya cathayensis* Sarg.) is an economically important nut tree in southeast China (Zheng et al., 2010; Li et al., 2014). Successful grafting may improve the quality and production of the hickory trees by enhancing nutrient absorption, stress tolerance, growth performance, and disease resistance in the graft units (Martínez-Ballesta et al., 2010). Therefore, understanding proteome dynamics is useful when exploring the molecular mechanisms involved in the grafting process.

Quantitative proteomic studies have quantified the changes in protein abundance to identify their biological functions (Alexandersson et al., 2013). LC-MS/MS is a new quantitative proteomics technique that has already been used in tree research over the past few years. The large number of proteins that have been identified suggests a deeper and more comprehensive analysis of distinct protein expression patterns should be conducted during the grafting process of hickory.

Callus formation is the first and basic step in a successful grafting process. Once the callus has formed, several other key events seem to be essential for the development of future vascular connections (Pina and Errea, 2005). To establish a new cellular homeostasis, the graft interfaces actively defend the graft against stress by modifying its physiological and proteomic responses. Oxidative stress in graft interfaces has been also reported (Irisarri et al., 2015). Proteomic analysis of tomato graft unions showed an increase in the activities of several antioxidant enzymes, such as superoxide dismutase (SOD), catalase (CAT), and ascorbate peroxidase (APX) (Muneer et al., 2016). Six differentially expressed antioxidant genes (SOD1, SOD3, APX3, APX6, CAT1, and CAT3) were identified in heterografts of pear/quince combinations (Irisarri et al., 2015). In *Arabidopsis*, overexpressing SOD, which catalyzes the dismutation of superoxide radicals, enhanced early callus induction and shoot regenerative capacity (Shafi et al., 2015). In our study, four APXs, three CATs, and six SODs were quantified in hickory during the grafting process. Among these antioxidant enzymes, a Cu-Zn superoxide dismutase and a Cu/Zn-superoxide dismutase copper chaperone precursor were significantly up-regulated (Gao et al., 2014). Higher SOD activity in 7 days grafting union than in the control might indicated that the H_2_O_2_ produced in excess by these two superoxide dismutases may promote the expression of several genes responsible for grafting union formation. Besides, three CATs and two APXs were significantly down-regulated during the grafting process, which could be associated with the changes in the protection of the tissue damage during grafting process. Our data suggested that grafting may generally affect the antioxidant defense systems in hickory.

Proteins related to ‘abiotic stimulus responses’ have also been reported to be involved in the grafting process. In watermelon, high light intensity plays an important role in the vascular connection and protein expression responses in grafted seedlings (Muneer et al., 2015). In tomato, grafting unions have varied the responses to temperature stress, and different rootstocks can affect the photosynthetic responses to drought (Muneer et al., 2016). Three stress response-related GO terms: GO:0009607, GO:0006950, and GO:0050896, were identified in hickory by enrichment analysis (**Figure 3**). Pathogenesis-related (PR) proteins, which can be grouped into 17 families and have antimicrobial activities, are elicited in many plant species after attack by different pathogens (van Loon et al., 2006). Interestingly, the protein levels of three PR family proteins identified in this study significantly increased during the hickory grafting process. In particular, PR10, a homolog of the major allergen Pru ar 1, was up-regulated 3.75 fold during the grafting process in plum. Our proteomic analysis highlighted the specific accumulation of PR10 proteins, which are essential for the survival of grafting unions in natural environments.

Auxin have various biological functions (Feng et al., 2015; Yu et al., 2015). Auxin signaling homeostasis has been reported to be involved in the plant graft process (Beveridge et al., 2000; Yin et al., 2012). In other species, the expression of several auxin-related genes is regulated during the graft process. For example, in grapevine, an auxin influx carrier encoding gene is significantly up-regulated in the graft interface zone during the early stage after grafting (Cookson et al., 2013). Auxin is essential for vascular tissue development (Ye, 2002). In hickory, four auxin response proteins were identified and quantified (**Figure 4C**). The changes in their expression levels suggested that the auxin signaling pathway may be involved in vascular establishment during the grafting process. In *Arabidopsis*, auxin canalizes the pathway to direct the reconnection of vascular tissue between the scion and rootstock (Yin et al., 2012). Increasing auxin levels can trigger the differentiation of xylem cells (Cano-Delgado et al., 2010; Matte Risopatron et al., 2010). For example, the highest levels of auxin are found in the cambium of *Pinus* and *Populus*, consistent with a role for auxin in maintaining cambial cell identity (Uggla et al., 1996). Interestingly, the formation of endogenous auxin content was also induced, confirming an important role of auxin in the pattern formation of vascular tissue during the grafting process of hickory.

The regulation of metabolisms, including the Calvin cycle, glycolytic pathway, energy metabolism, and reactive oxygen metabolism, increase the biomass and photosynthetic capacity of graft-compatible cucumbers (Xu et al., 2016). In grafted grapevines, nearly half of the transmitting genes observed in *in vitro* grafts (47.4%) were related to the ‘cellular metabolic process’ (Yang et al., 2015). The GO term classification suggested that the DEPs were probably associated with ‘catalytic activity’ (41.0%) and ‘metabolic process’ (40.0%), suggesting that metabolism was involved in the hickory grafting responses (**Figure 2A**). Previous reports have shown that a number of antioxidant secondary metabolites were formed simultaneously during the rapid growth phase of callus cultures (Luo et al., 2003; Zhang et al., 2009; Maneechai et al., 2012). For example, flavanone accumulated at the union of *Prunus* grafts (Treutter and Feucht, 1988). Genes involved in flavonoid biosynthesis were also up-regulated at the graft interface in grapevine (Cookson et al., 2013). Among the enriched metabolism pathways, the ‘flavonoid biosynthesis’ pathway was significantly up-regulated during the grafting process of hickory. Interestingly, five important enzymes: flavanone 3-hydroxylase, a cinnamate 4-hydroxylase, a dihydroflavonol-4-reductase, a naringenin-chalcone synthase, and a chalcone isomerase, were significantly up-regulated (**Figure 5B**). Further investigation verified that a significant increase occurred in the total flavonoid content in the scions (**Figure 6A**), which suggested that graft union formation increases the content of a series of downstream antioxidant secondary metabolites by activating flavonoid biosynthesis. A number of studies have reported that a close relationship exists between the production of antioxidant secondary metabolites and callus formation (Fazal et al., 2016a,b). The accumulation of flavonoids may positively affect the grafting process by enhancing callus formation. Additionally, activation of flavonoid biosynthesis in plants is associated to sensitivity to environmental stresses (Zandalinas et al., 2016). After cutting, an induction of antioxidant flavonoid biosynthesis was observed in potatoes (Tudela et al., 2002). Increasing flavonoid biosynthesis may play an important role in the protection of the tissue damage during grafting process.

## Conclusion

In this study, a proteomics-based approach was used to reveal differential protein profiling during the hickory grafting process. A large number of DEPs were identified and analyzed based on their major biological functions. The KEGG analysis showed that proteins related to the flavonoid biosynthesis pathway were significantly up-regulated. Furthermore, a significant increasing in the total flavonoid contents was observed in the scion. The accumulation of flavonoids may play a role in the callus formation during grafting process.

## Author Contributions

YT, DX, LQ, LZ, and HY carried out the molecular studies, participated in the analysis and drafted the manuscript. WG carried out the qRT-PCR analysis. HL performed the statistical analysis. DY and BZ conceived of the study, and participated in its design. BZ acquired of funding. CS helped to draft the manuscript. All authors read and approved the final manuscript.

## Conflict of Interest Statement

The authors declare that the research was conducted in the absence of any commercial or financial relationships that could be construed as a potential conflict of interest.

## Footnotes

1 http://www.matrixscience.com/

2 http://www.ebi.ac.uk/GOA/

3 http://www.genome.jp/kegg/

4 http://www.genome.jp/tools/kaas/

5 http://www.kegg.jp/kegg/mapper.html

6 http://www.ebi.ac.uk/interpro/

7 http://www.dikma.com.cn
