# Supplementary material for: Comparative Proteomic Analysis of the Graft Unions in Hickory (Carya cathayensis) Provides Insights into Response Mechanisms to Grafting Process
Source: Front Plant Sci. 2017 Apr 27;8:676. doi: 10.3389/fpls.2017.00676 (PMC5406401; doi:10.3389/fpls.2017.00676)
Supplement: Supplementary file 7 [file Image_1.pdf]

Figure S1 The detail information of sampling and histochemically checking for callus formation.

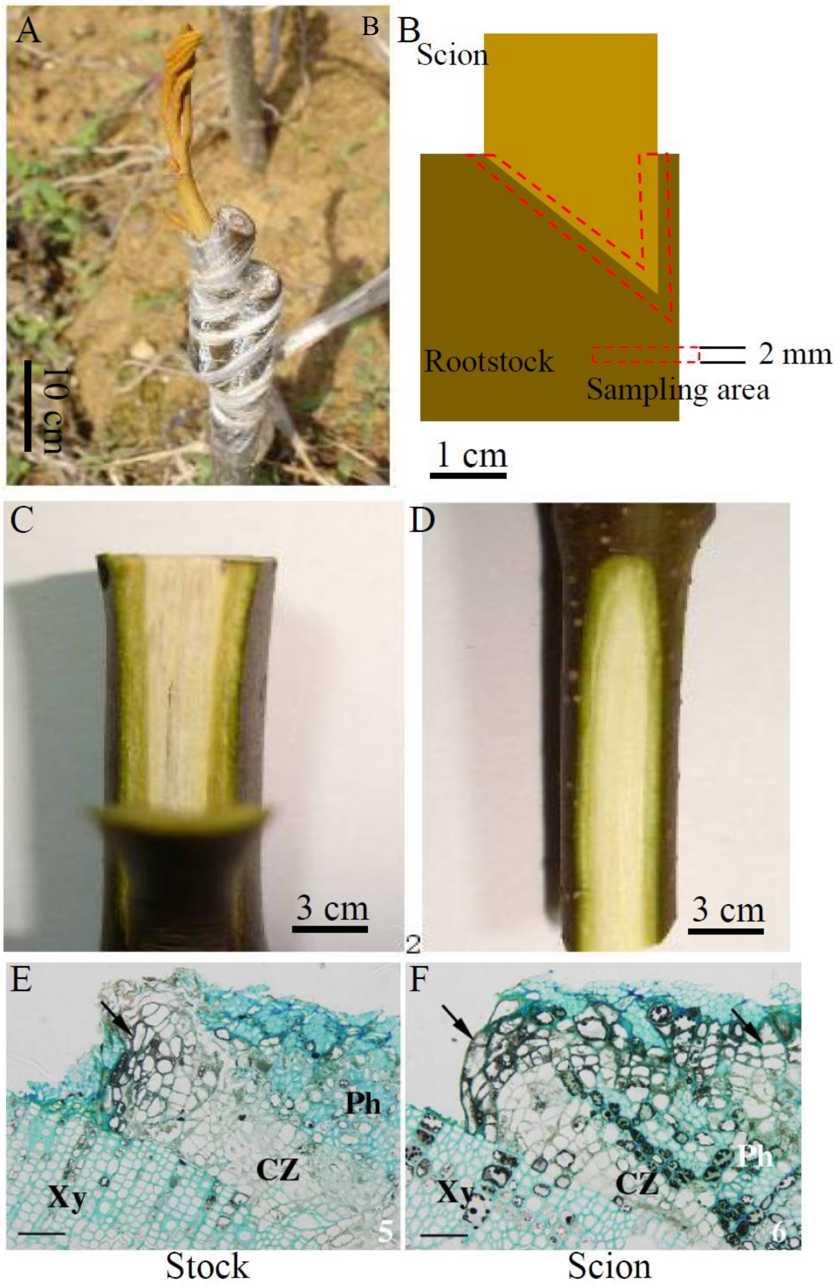

Fig. S1 The detail information of sampling and histochemically checking for callus formation. (A) A photograph of the hickory grafting process. Bar = 10 cm (B) A hand drawn picture for the sampling of our grafting experiment. Bar = 1 cm (C) Rootstock and (D) scion for our grafting experiment. Bar = 3 cm. The histochemically checking for callus formation at scion (E) and stock (F) during the hickory grafting process. CZ: Cambium zone； Ph: Phloem; Xy: Xylem. Arrow indicated differentiated callus in grafting face. Bar = 100 µm.
