# Supplementary material for: Comparative Proteomic Analysis of the Graft Unions in Hickory (Carya cathayensis) Provides Insights into Response Mechanisms to Grafting Process
Source: Front Plant Sci. 2017 Apr 27;8:676. doi: 10.3389/fpls.2017.00676 (PMC5406401; doi:10.3389/fpls.2017.00676)
Supplement: Supplementary file 8 [file Image_2.PDF]

Figure S2

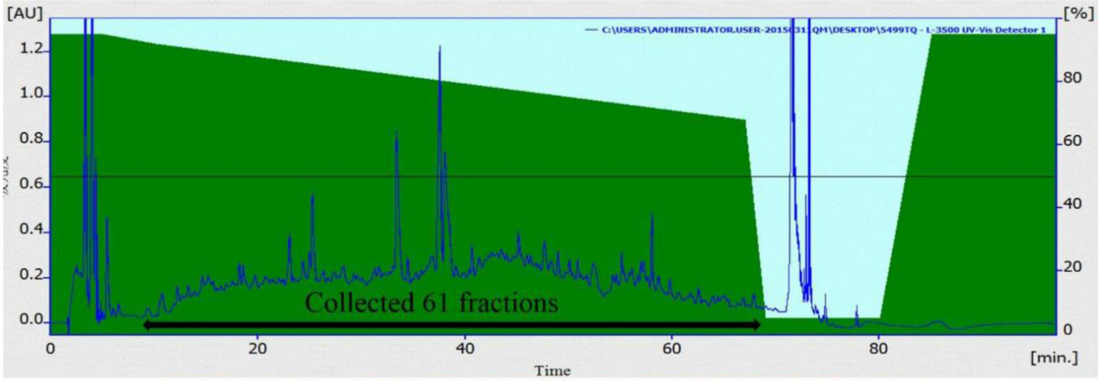

1	2	3	4	5	6	7	8	9	10	11	12	13	14	15	16	17	18
19	20	21	22	23	24	25	26	27	28	29	30	31	32	33	34	35	36
37	38	39	40	41	42	43	44	45	46	47	48	49	50	51	52	53	54
55	56	57	58	59	60	61											

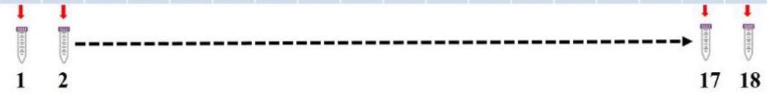

Fig.S2 A representative HPLC data. The 61 fractions during 61 min gradient were pooled to 18 final fractions for mass spectrometry analysis.
