# Supplementary material for: Comparative Proteomic Analysis of the Graft Unions in Hickory (Carya cathayensis) Provides Insights into Response Mechanisms to Grafting Process
Source: Front Plant Sci. 2017 Apr 27;8:676. doi: 10.3389/fpls.2017.00676 (PMC5406401; doi:10.3389/fpls.2017.00676)
Supplement: Supplementary file 9 [file Image_3.PDF]

Figure S3

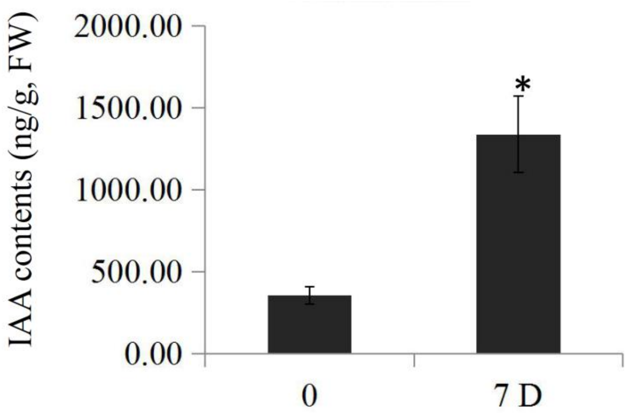

Fig. S3 The IAA contents in the grafting union during the hickory grafting process. “*” indicated significantly differences in IAA contents between 0 and 7 D.
